# Testicular Abscess an Unusual Cause for Febrile Neutropenia

**DOI:** 10.1100/tsw.2008.135

**Published:** 2008-10-03

**Authors:** Tal Grenader

**Affiliations:** Department of Oncology, Shaare Zedek Medical Center, Jerusalem, Israel

**Keywords:** testicular abscess, testicular cancer, neutropenic fever, chemotherapy, orchiectomy, BEP

## Abstract

Patients with good-risk disseminated testicular cancer are effectively managed with platinum-based chemotherapy. Febrile neutropenia is a dose-limiting event for many chemotherapy regimens. The risk of developing febrile neutropenia is related both to the chemotherapy dose and schedule, and to patient-related factors. Among patients who require ongoing chemotherapy for metastatic disease, it is very unusual for surgical complications to delay the initiation of chemotherapy. We describe a patient who developed febrile neutropenia with testicular abscess when treated with BEP 2 weeks following inguinal orchiectomy.

## INTRODUCTION

Testicular cancer is the most common malignancy in men aged 20–34 years and several publications have reported a major increase in the incidence of testicular cancer in recent decades[1]. Patients with good-risk disseminated disease are effectively managed with three courses of BEP (bleomycin, etoposide, cisplatin) or four courses of a two-drug combination EP.

The preferred operation for suspected testicular cancer is radical inguinal orchiectomy with ligation of the spermatic cord at the level of the internal ring. Postoperative wound hemorrhage is the most common complication following surgery, and may result in scrotal or retroperitoneal hematomata[2,3]. Infection is rare among patients who require ongoing chemotherapy for disease; it is very unusual for surgical complications to delay the initiation of chemotherapy.

We describe a patient who developed febrile neutropenia with testicular abscess when treated with BEP 2 weeks following inguinal orchiectomy.

## CASE HISTORY

A 26-year-old man with moderate mental retardation noticed a large left testicular mass and was admitted to the urology department of Shaare Zedek Medical Center. On examination, he was found to have a nontender left testicular mass of 3–4 cm. The right testicle was normal. Laboratory data showed levels of serum alpha-fetoprotein of 480 ng/mL (reference range: 0–15), beta-human gonadotropin of 209 mIU/mL

(normal <5), and lactase dehydrogenase of 939 U/L (reference range: 313–618), respectively. Chest radiography revealed multiple bilateral pulmonary cannonball-type lesions too numerous to count. Computerized tomography (CT) of the chest confirmed this finding. CT of the abdomen and pelvis revealed massive periaortic and pericaval lymphadenopathy from the level of the left renal vein extending to the aortic bifurcation and right-sided hydronephrosis. CT of the head was normal.

The patient underwent left radical inguinal orchiectomy. The pathological diagnosis of his testicular tumor was that of an embryonal carcinoma. One week following the operation, chemotherapy with BEP protocol was commenced.

Ten days after the initiation of therapy, the patient presented with rigors, fever, abdominal pain, and general weakness. On admission, his temperature was over 39.0°C. He had minimal abdominal tenderness in the left lower quadrant without peritoneal irritation. There was slight swelling, redness, and tenderness of the scrotum, but his inguinal wound was healing normally with no evidence of inflammation or exudation. Laboratory investigations revealed a WBC of 800/mm³ and 28% neutrophils. Recombinant granulocyte colony-stimulating factor (G-CSF) (filgrastim), ceftazidime, and gentamyicin were given empirically for febrile neutropenia for 4 days, under which regime the white cell count rose to 5700/mm³. The lower abdominal pain and tenderness, and the fever (over 38.5°C), did not resolve. Blood and urine cultures were positive for *Klebsiella pneumonia* and *Escherichia coli*.

Re-examination of the scrotum revealed the development of diffuse erythema and exquisite tenderness, predominantly on the left side. Ultrasound of the scrotum showed a complex collection with internal septa. The clinical and ultrasound findings suggested a diagnosis of epididymal abscess. The collection was aspirated with a 14-gauge needle, with removal of 60 cc of pus, and the patient subsequently underwent scrotal exploration and drainage of the abscess. This resulted in rapid resolution of his symptoms. Cultures yielded *K. pneumonia* and *E. coli*. The patient was discharged 7 days later.

He subsequently completed a further two cycles of BEP chemotherapy with filgrastim support without further infectious complications.

## DISCUSSION

Febrile neutropenia is a dose-limiting event for many chemotherapy regimens. The risk of developing febrile neutropenia is related both to the chemotherapy dose and schedule, and to patient-related factors. In addition to its impact on quality of life, febrile neutropenia predisposes patients with cancer to serious and often life-threatening infections.

In the absence of respiratory distress, there is no urgency to initiate chemotherapy within 1 or 2 days following orchiectomy, and our usual practice in such cases would be to commence the BEP regimen after 2–3 weeks. While the patient described had no respiratory distress, in view of his disseminated disease, it was considered prudent to commence treatment just 1 week following his operation. The BEP regimen is associated with substantial hematological toxicity, with an incidence of 34% grade 4 neutropenia and of infectious death of up to 2%[4]. In a placebo-controlled study, the routine use of filgrastim from the first of six cycles of BEP or BOP/VIP-B regimens significantly improved the delivery of the planned treatment schedule without effect on either failure-free or overall survival REF. The use of filgrastim was associated with a clinically important reduction in the number of toxic deaths, confined to the experimental BOP/VIP-B regimen. The rate of febrile neutropenia in the placebo group was 29.5%, compared with19.5% in the filgrastim group[5].

In general, patients with febrile neutropenia are evaluated for a possible site of infection and are treated with antibiotics. The need for admission and intravenous antibiotics and neutrophil CSFs is determined on clinical grounds in accordance with contemporary guidelines[6]. Severe neutropenia may mask signs of infection and, often, the site of infection may only become clinically manifest after neutrophil recovery. An additional factor that may have contributed to the development of an abscess in this patient was his mental state, which led him to touch and probe the region of the wound.

## CONCLUSION

In general, this case highlights the need for a high level of clinical suspicion and clinical vigilance for the potential of wound infection among recent surgical patients with febrile neutropenia. This case specifically highlights the need to consider scrotal abscess in patients with febrile neutropenia following recent orchiectomy.
